# BENEFICENCE, COMPANIONSHIP AND FINANCES AS MOTIVATORS FOR PARTICIPATION IN INTERGENERATIONAL HOME SHARING

**DOI:** 10.1093/geroni/igz038.3489

**Published:** 2019-11-08

**Authors:** Cary Sweeney, Rachel K Bell

**Affiliations:** University of California, Berkeley Retirement Center, Berkeley, California, United States

## Abstract

In January 2019, the UC Berkeley Retirement Center began piloting Berkeley Home Match (BHM), a program that matches graduate students who need affordable housing with retirees who live near campus and have an extra room. BHM seeks to address affordable housing challenges for retirees and students while creating meaningful relationships. While some data exists for home sharing programs, less is known about the motivators and outcomes of retirees and students participating in these programs. In addition to decreasing the financial burden of housing, Social Exchange Theory suggests the built-in reciprocity of intergenerational home sharing may facilitate meaningful relationships. The purpose of this program evaluation was to understand the motivating factors for participation in a University-based home sharing program. Applicants (N=35) rated factors that influenced their decision to participate using a 7-point scale (1=“Not Influential”, 7=“Very Influential”). On average, homeowners (N=6) rated “helping a student” 5.2, “income” 5.2, and “companionship” 3.6, while home seekers (N=29) rated “helping an older adult” 5.03, “rent price” 6.1, and “companionship” 4.76. Surprisingly, ratings for “helping a student” and “helping a senior” were the same or close to ratings for “income” and “rent prices,” suggesting beneficence could be a key motivator and interdependence a mechanism for relationship development. Additionally, students’ rating for “companionship” was higher than expected, indicating an openness to the relationship. These findings as well as other factors impacting home matching will be discussed, including participant demographics, reduced housing cost, housing characteristics, and long-term program feasibility.

